# Poverty and Childhood Obesity: Current Evidence and Methodologies for Future Research

**DOI:** 10.1007/s13679-025-00627-x

**Published:** 2025-04-11

**Authors:** Richard Liang, Ryunosuke Goto, Yusuke Okubo, David H. Rehkopf, Kosuke Inoue

**Affiliations:** 1 Department of Epidemiology and Population Health, Stanford University School of Medicine, Stanford, CA USA; 2 Department of Biomedical Data Science, Stanford University School of Medicine, Stanford, CA USA; 3 Department of Social Medicine, National Center for Child Health and Development, Tokyo, Japan; 4 Center for Population Health Sciences, Stanford University School of Medicine, Stanford, CA USA; 5 Department of Health Policy, Stanford University School of Medicine, Stanford, CA USA; 6 Department of Medicine, Stanford University School of Medicine, Stanford, CA USA; 7 Department of Pediatrics, Stanford University School of Medicine, Stanford, CA USA; 8 Department of Sociology, Stanford University, Stanford, CA USA; 9 Department of Social Epidemiology, Graduate School of Medicine, Kyoto University, Floor 2, Science Frontier Laboratory, Yoshida-konoe-cho, Sakyo-ku Kyoto, Kyoto, 604-8146 Japan; 10 Hakubi Center for Advanced Research, Kyoto University, Kyoto, Japan

**Keywords:** Childhood Obesity, Poverty, Socioeconomic Status, Quasi-experimental Studies, Causal Inference

## Abstract

**Purpose of Review:**

This narrative review summarizes current knowledge on the link between poverty and childhood obesity, and then describes conventional and modern epidemiologic methods for causal inference that may help provide more robust evidence on how poverty reduction can prevent childhood obesity.

**Recent Findings:**

Household poverty has been consistently associated with increased risk of childhood obesity across observational studies in industrialized countries. Due to ethical and feasibility limitations, few randomized controlled trials directly test the effect of poverty reduction. A growing number of studies use quasi-experimental methods to study the effects of poverty reduction policies on childhood obesity. These methods include instrumental variables, difference-in-differences, interrupted time series analysis, and regression discontinuity. Other complementary methods such as causal mediation analysis allow us to elucidate the mechanisms of how poverty reduction affects childhood obesity outcomes, while examining heterogeneous treatment effects using cutting-edge machine learning algorithms may further identify subpopulations that benefit the most from poverty interventions.

**Summary:**

Despite the strong associations between poverty and childhood obesity observed in industrialized countries, current evidence about the causal effect of poverty reduction on childhood obesity is mixed. This is likely due to the complex etiology of childhood obesity and potentially unintended effects of policies. Future studies that leverage advances in causal inference with quasi-experimental approaches will help provide more robust evidence to help guide practitioners and policymakers in ongoing childhood obesity prevention efforts.

## Introduction

Population levels of obesity are increasingly recognized as an urgent global public health concern [1–3]. In particular, the prevalence of obesity during childhood and adolescence has been rising at an alarming rate, with growing evidence that childhood and adolescent obesity increases the risk of premature morbidity and mortality during young adulthood even before the age of 30 years [4]. With such notable health risks and increasing prevalence attributed to this condition, multiple approaches must be taken to attain the World Health Organization’s 2025 Global Nutrition Target of no increase in childhood overweight [5].

Despite increasing knowledge of how upstream factors, such as government policies and socioeconomic conditions, contribute to childhood obesity, there has been a disproportionate focus on interventions that target downstream determinants of health, such as clinical treatments and individual-level behavioral interventions [6]. Targeting wider, upstream social determinants of health through policy and programmatic approaches is critical to moving the needle on addressing childhood obesity [7, 8]. Of these social determinants of health, socioeconomic status and poverty, in particular, have substantial consequences on the development of childhood obesity [9], which received notable attention in the USA in the National Academies of Sciences, Engineering and Medicine report in 2019 emphasizing the importance of poverty reduction in improving child health and well-being [10, 11].

The purpose of this narrative review is to summarize the state of childhood obesity and describe current knowledge on the link between poverty and childhood obesity. This paper will also introduce specific methods for causal inference to promote further research on whether and how reducing poverty can prevent childhood obesity, as there are growing calls for clinicians and epidemiologists to leverage advances in causal inference to build upon non-causal associational studies and push towards estimating causal effects of interest [12–15].

## The Current State of Childhood Obesity

### Definitions of Childhood Overweight and Obesity

The World Health Organization (WHO) defines overweight as a condition of excess fat deposits and obesity as a chronic disease characterized by excess fat deposits that can impair health [16]. To define these conditions among children, the WHO uses the WHO Child Growth Standards for children under 5 years of age and the WHO Growth Reference for children between 5 and 19 years of age. For children under 5 years of age, overweight and obesity are defined as weight-for-height (or weight-for-length for children under 2 years of age) greater than 2 and 3 standard deviations, respectively, above the median for the 2006 WHO Child Growth Standards; for children between 5 and 19 years of age, overweight and obesity are defined as greater than 1 and 2 standard deviations, respectively, above the median for the 2007 WHO Growth Reference [17]. For surveillance and epidemiological purposes, body mass index (BMI) is often calculated as a proxy for excess fat deposits. For brevity, we use the term childhood obesity to generally refer to the conditions of overweight and obesity in children under the age of 18 years old.

### Epidemiology

Childhood obesity has been increasing in prevalence around the world, with a sharp 1.5-fold increase from 2012 to 2023 compared to 2000–2011 [18]. The global pooled prevalence of children who are overweight and obese from 2000 to 2023 is 14.8% and 8.5%, respectively. There is also considerable geographical variation, with higher prevalences of obesity in more developed regions and higher-income countries. Furthermore, the World Obesity Federation projects that by 2035, two in five children globally will be living with being overweight or obese, accounting for over 750 million children between ages 5–19 years [19]. In addition to prevalence, the incidence of childhood obesity has also been increasing. In a study of kindergarten cohorts in the United States, childhood obesity has been occurring at higher rates at younger ages and more severity, with a 4.5% relative increase from a 15.5% cumulative incidence in 1998, to a 16.2% cumulative incidence in 2010 [20].

Socioeconomic disparities in obesity have also been increasing. For example, Goto et al. reported a widening socioeconomic disparity in adolescent obesity prevalence in the United States, with the difference in prevalence increasing by 6.4% from 1999 to 2018 by household income stratifications (≤ 138% vs. > 138% federal poverty level) [21]. Moreover, childhood obesity prevalence in the United States is higher among racial/ethnic minority children [22]. In a study comparing 2011 to 2012 with 2017 to 2020, childhood obesity prevalence increased for Mexican American and non-Hispanic Black children, but not for non-Hispanic White children [23]. Maternal education has also been linked to childhood obesity. In one study of six high-income countries, social gradients by maternal education on the risk of childhood obesity were identified across all study cohorts, in which lower maternal education was associated with childhood overweight and obesity at ages 8–11 years [24]. However, it is important to note that the relationship between different aspects of socioeconomic status, such as educational attainment, and obesity also depends on a given country’s level of development [25–27].

There are several potential mechanisms that lead to childhood obesity. The most prominent model for conceptualizing the pathogenesis of obesity is the energy balance model, which implicates dysregulation of energy intake and expenditure in the development of obesity [28]. Different pathways can lead to obesity by influencing excess energy intake and decreased energy expenditure, such as mechanisms that affect the quality and quantity of diet and physical activity, or other mechanisms that modulate metabolic and physiological processes in obesity pathogenesis. The bio-socioecological framework has been proposed to connect these various etiologies and explain the rise of childhood obesity around the world [29]. The bio-socioecological framework posits that individual biological predisposition, socioeconomic conditions, and built environmental factors work together to affect the risk of childhood obesity [30–32]. In 2007, the United Kingdom government’s Foresight Programme identified 108 of such variables and conceptualized them into an “obesity systems map” [33]. Since then, other researchers have applied the Foresight model to childhood obesity and highlighted the need for multisector and multidisciplinary approaches to tackling the challenge of childhood obesity [34, 35]. This review paper focuses on poverty as a socioeconomic etiology of childhood obesity, with most currently available evidence being limited to high-income country contexts.

### Consequences of Childhood Obesity

Childhood obesity has numerous health consequences, psychosocial effects, and economic burdens. Obesity affects children through both short-term and long-term health effects, with short-term health challenges including increased risk for severe outcomes in hospitalized children with asthma exacerbations and respiratory infections [36–38]. Long-term health complications of childhood obesity include increased risk of cardiovascular disease, respiratory conditions, and endocrine dysfunction in adulthood, with some risks increasing even later in childhood [29]. Psychosocial effects include increased risk of stigma, bullying, fatigue, depression, anxiety, and eating disorders. There are also major economic implications of childhood obesity, with direct and indirect annual healthcare costs projected to reach about $13.6 billion and $49.0 billion, respectively, by 2050 across the world (in 2022 US $) [39].

Given the numerous health consequences, psychosocial effects, and economic burdens described above, it is important to address the key drivers of childhood obesity. In particular, given the bidirectional relationship between socioeconomic status and childhood obesity, addressing this pressing public health issue from a social epidemiology perspective is imperative not only to improve individual children’s health, but also to avoid the cycle of obesity and poverty across generations [40]. For example, one study from Spain comparing families in 2003–2004 and 2006–2007 estimated that obese parents had a 4–5% increased probability of having obese children [41]. The researchers estimated that income-related inequality in childhood obesity (as measured by concentration indices) increased by 50% during the study period, and that parental obesity accounted for 6–12% of this income-related inequality.

In adulthood, lower income has been associated with subsequent obesity, with one meta-analysis reporting a pooled odds ratio of 1.27 (95%CI: 1.10–1.47) and risk ratio of 1.52 (95%CI: 1.08–2.13), although after adjusting for publication bias, the estimates were attenuated and no longer statistically significant [42]. The same meta-analysis examined reverse causation and found a consistent association between obesity and subsequent income, with a standardized mean difference of − 0.15 (95%CI: −0.30 to -0.01), even after accounting for publication bias. These findings suggest that income and obesity may be mutually reinforcing, although more evidence is needed to better understand income-related inequalities in obesity. Potential mechanisms that connect childhood obesity to this cycle include human capital development. For instance, there is evidence from high-income countries that childhood obesity hinders cognitive performance, educational attainment, and labor market outcomes [43], as well as executive functioning skills [44]. Deficits in such components in human capital development may affect potential income earnings, thus contributing to the cycle of poverty and adverse health outcomes from one generation to the next.

### The Link between Poverty and Childhood Obesity

Poverty has profound effects on child health. However, while low-income countries have overall lower rates of childhood obesity as compared to high-income countries [18], children living in poor communities within high-income countries paradoxically have the highest rates of obesity [45]. Therefore, researching the question of whether reducing poverty prevents childhood obesity depends on regional context and the measurement level of poverty.

### Definitions of Poverty

There are several ways to define poverty. For example, the WHO Nutrition Landscape Information System tracks the proportion of the population below the international poverty line, defined as living on less than US$1.90 a day at 2011 international prices [46]. Meanwhile, in the United States, federal poverty levels are set based on family size and are used to determine eligibility for reduced-cost health coverage (e.g., income below 138% of the federal poverty level confers eligibility for Medicaid, a free or low-cost governmental health insurance plan) [47]. In addition, there may be differences between poverty measured at the individual- or household-level and poverty measured at the neighborhood- or area-level [48]. When researching whether poverty reduction prevents childhood obesity, there are also several ways to define “poverty reduction.” For example, does one attempt to eliminate poverty completely in a given area, or does one attempt interventions such as cash transfers or tax credits to reduce the severity of poverty among individuals [49]? These are all important considerations when identifying the link between poverty (broadly defined) and childhood obesity.

### What Is Known about Poverty and Childhood Obesity?

The associations between poverty and childhood obesity are well documented by numerous observational studies. For example, children born into poverty or who were intermittently poor during early childhood in the Netherlands were found to be at higher risk for overweight and obesity at 6 years of age, as compared to those not born into poverty [50]. This result is congruent with findings from the United States, where recurrent household poverty conferred the greatest risk of childhood obesity [51]. In the United Kingdom, children living in persistent poverty since birth were at higher risk for obesity at age 14 years, as compared to those in low poverty and adversity [52]. Meanwhile, children in Canada who are consistently poor or have an increasing risk of being poor have an increased risk of being overweight/obese at ages 8, 10, and 12 years [53]. In terms of a broader definition of poverty, material deprivation (defined as both poverty and parental unemployment) was associated with slightly increased BMI among children in Denmark [54].

While the observational studies described above show a consistent link between poverty and childhood obesity, there have been more mixed results in interventional and quasi-experimental studies that seek to answer causal questions of whether reducing poverty improves childhood obesity or not. For instance, a cash transfer program intervention for low-income families in Mexico was observed to have modest effects on reducing BMI and the risk of being overweight among children [55]. Among quasi-experimental studies, one study found that opening or expanding casinos in American Indian tribal lands was associated with decreased poverty and decreased risk of childhood overweight and obesity using a difference-in-differences approach [56]. In a study that used the Earned Income Tax Credit (EITC) policy as an instrumental variable, increases in net income for eligible low-income families have been associated with improvements in various child health outcomes in the United States [57]. However, one study reported an opposite finding, that the EITC policy actually slightly increased the risk of childhood obesity [58]. The author explained this counterintuitive finding by suggesting that mothers who were eligible for EITC were also more likely to find employment opportunities and spend more time at work, which may have led to less time directly caring for their child’s diet or physical activity.

*Proposed Mechanisms Between Poverty and Childhood Obesity*.

**Fig. 1:**
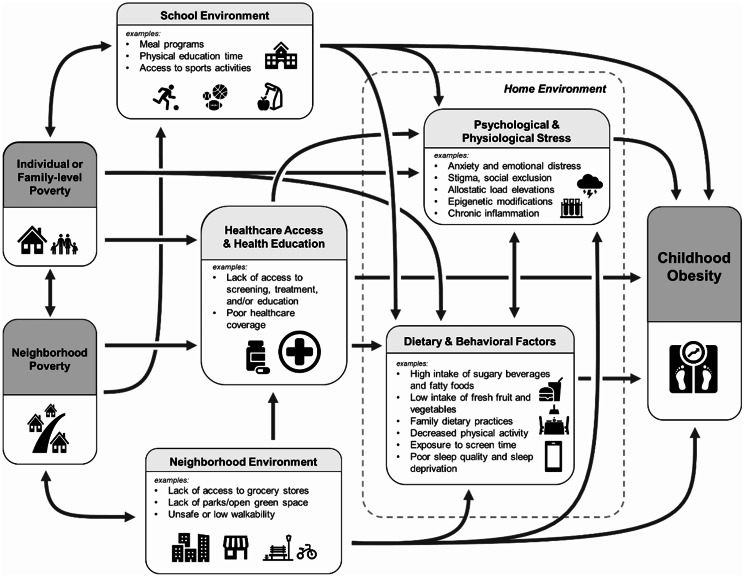
Conceptual Map of the Link Between Poverty and Childhood Obesity

Figure 1 depicts a conceptual map of the various factors connecting poverty and childhood obesity. Low income affects the availability and affordability of food, with foods high in calories, fats, and sugars typically costing less than high-quality, nutrient-dense foods [45]. It is important to note that our conceptual map primarily applies to high-income countries because evidence from low- and middle-income countries is currently limited, and the underlying mechanisms may vary between these regions. For example, in low-income country contexts, additional discretionary income in those contexts may decrease energy expenditures of manual labor-intensive jobs and increase surplus consumption of calorie-dense foods, thus leading to an increased risk of obesity [26, 59].

Individual- or family-level poverty also impacts the type of neighborhood one can live in, with impoverished neighborhoods often lacking access to grocery stores, safe and open spaces for physical activity, and school environments with nutritious food and physical education programs [9]. These factors at the individual- and neighborhood-level influence behavioral factors, such as family dietary practices and intake of sugary food and beverages, that increase the risk of childhood obesity [60]. An individual’s interpersonal social environment may also contribute to the risk of obesity, in which weight gain in one individual may influence behaviors and tolerance for weight gain among their social contacts [61].

Moreover, the constant psychological distress and adverse childhood experience of living in poverty lead to the accumulation of toxic stress. This long-term exposure to stress is thought to increase the risk of obesity through several mechanisms, including sustained elevations in allostatic load [62], epigenetic modifications [63], and chronic activation of the hypothalamic-pituitary-adrenal axis [64].

*What is Unknown about Poverty and Childhood Obesity?*

Despite the substantial progress on knowledge about the relationship between poverty and childhood obesity, much remains unknown. As suggested by previous literature [58], there may be unintended consequences of poverty reduction policies that counteract beneficial effects on childhood obesity prevention. In addition, although several pathways have been suggested to link poverty and childhood obesity (as described in Fig. 1), how each mechanism affects the risk of childhood obesity is still not well-established. Poverty can also affect child nutrition status through the double burden of undernutrition and overnutrition [65]. On one hand, poverty can lead to undernutrition and reduced obesity risk due to an overall lack of food, but on the other hand, it can increase the risk of obesity when unhealthy, calorie-dense foods become the alternative to a lack of food. This heterogeneity of both decreasing and increasing obesity risk may then affect the interpretation of study results. Most evidence also appears to come from high-income countries, and there is still a gap in knowledge about how poverty and childhood obesity are linked in low- and middle-income countries. A deeper understanding of poverty dynamics in low- and middle-income countries is critical for social epidemiologists and other health researchers to generate socially useful evidence on ways to address childhood obesity and various other global health challenges [66, 67].

There are several challenges to studying these unknowns about poverty and childhood obesity. One major issue is the variability in how studies define poverty as an exposure, so caution must be taken to ensure that the consistency assumption is met when studying poverty and childhood obesity. This assumption requires that a given exposure variable is defined with enough specificity such that there are no multiple versions of the exposure variable hidden within the given definition [68]. For instance, the health effects of moving a family from a high-poverty neighborhood to a low-poverty neighborhood may differ from the health effects of improving that family’s current neighborhood from high-poverty to low-poverty [49]. While both scenarios represent “poverty reduction,” the different ways in which poverty is reduced may yield different health effects, therefore potentially violating the consistency assumption.

Poverty is also one of many components of social risk, and many studies use a composite measure of socioeconomic status. For example, various social disadvantage indices operationalize poverty differently and may have different implications on the risk of childhood obesity [69]. Such composite measures make it difficult to parse out the effect of poverty itself. There is also considerable overlap and correlation between poverty and other childhood environment such as adverse childhood events, further complicating the identification of the effect of poverty itself [70].

## Methodologies to Study Poverty and Childhood Obesity

To address these challenges, researchers can apply some conventional and modern causal inference methodologies that have increasingly received attention in medical, epidemiological, and health policy research [12–15], although this is not to minimize the real value and importance of descriptive research that are foundational to characterize the determinants and distribution of health and disease [71, 72]. In this final section, we review three broad types of questions that advance knowledge on the relationship between poverty and childhood obesity: (1) descriptive questions, (2) causal questions, and (3) questions of mechanisms.

### Descriptive Questions

Descriptive research questions are key to monitoring the distribution of health states in populations across time and place, as well as identifying relevant harmful or beneficial exposures. Descriptive studies not only help generate hypotheses and identify potential causal mechanisms, but also play an important role in disease surveillance and public health awareness. These types of observational studies can be cross-sectional, such as surveying a population at a given time point [18], or longitudinal, such as following individuals of a certain population over time [50, 52]. However, sometimes conducting such surveys at larger scales over long periods of time may be too cost-prohibitive or labor-intensive.

Pressing public health questions like childhood obesity requires urgent attention to find timely and effective solutions. In such cases, data linkages can be used to create powerful observational datasets that combine sources such as various government registries, administrative insurance databases, and/or electronic medical records to conduct descriptive research on childhood obesity [73]. For example, Elsenburg et al. were able to study the relation between material deprivation (including poverty) and BMI z-scores by using Danish registry data that linked school health examination results from one municipality with a nationwide register that covers all children born in Denmark [54]. Other studies in the United States have linked electronic health records with government census data to study the associations between area-level measures of poverty and childhood obesity [74, 75].

### Causal Questions

In contrast to descriptive research questions, causal questions seek to answer whether an outcome differs if a treatment, exposure, or policy is given versus withheld [76]. Such questions may be answered through interventional or quasi-experimental study designs. Table 1 summarizes several study methodologies that can be used to further advance knowledge on causal relations between poverty and childhood obesity prevention.

**Table 1:** Summary of study design to investigate causal relations between poverty reduction and childhood obesity

Study Design	Analytical Approach	Brief Summary of Approach	Examples of Potential Research Questions	Example of Application Papers
Interventional study	Randomized Control Trial and Non-Randomized Trial	Assign intervention to a treated and control group, then compare outcomes between groups to assess the effect of intervention.	What is the effect of a cash transfer program for low-income families on risk of childhood obesity?	Fernald et al. 2008 [55]; Ludwig et al. 2011 [77]
Observational study (Quasi-experimental study)	Instrumental Variable (IV)	Use a variable that is associated with the outcome only through the exposure as an instrument to estimate the causal effect of the exposure on the outcome.	What is the effect of a tax credit policy for low-income families on childhood obesity risk?	Jo 2018 [58]; Hamad & Rehkopf 2016 [57]
	Difference-in-Differences (DiD)	Compare longitudinal outcomes before and after the treatment (intervention or policy) was implemented between groups with or without treatment under the parallel trend assumption.	What is the effect of opening or expanding a casino in low-income American Indian tribal lands on childhood obesity outcomes?	Jones-Smith et al. 2014 [56]; Rummo et al. 2023 [85]; Localio et al. 2024 [86]; Hamad et al. 2018 [105]
	Interrupted Time Series (ITS)	Compare longitudinal outcomes before and after the treatment (intervention or policy) was implemented in a single group, accounting for over-dispersion autocorrelation, and time-varying confounders.	What is the effect of a school food nutrition policy on childhood obesity outcomes over time?	Chandran et al. 2023 [90]
	Regression discontinuity design (RDD)	Compare outcomes between individuals who are just above or just below a threshold for receiving treatment.	What is the effect of a food program for low-income schools on fresh fruit and vegetable intake among students?	Olsho et al. 2015 [92]

Interventional studies include randomized controlled trials, as well as non-randomized experimental studies. These types of studies typically involve assigning a certain treatment to a treated group and comparing outcomes with a control group. For instance, Fernald et al. assessed the effects of a conditional cash transfer program on child health outcomes, including childhood obesity [55]. In another example, Ludwig et al. studied the effects of a randomized social experiment in which women were assigned to receive housing vouchers (i.e., an opportunity to move from a high-poverty to a low-poverty neighborhood) and examined adult BMI differences, but did not examine childhood obesity specifically [77]. While this randomized social experiment, known as the Moving To Opportunity for Fair Housing Demonstration Project (MTO), was originally designed to focus on economic outcomes, the MTO is now a well-cited example of how moving to a low-poverty neighborhood has beneficial effects on obesity, diabetes, and other aspects of individual health and well-being [78, 79].

However, interventional studies may not always be appropriate or feasible to study whether poverty reduction prevents childhood obesity. For example, it is not ethical to assign impoverished conditions to people, or it may be too logistically challenging to run a large-scale trial that is nationally representative. In such cases, observational quasi-experimental studies may provide valuable evidence in situations where interventional studies are not possible. Quasi-experimental studies include methods such as instrumental variables, difference-in-differences, interrupted time series, and regression discontinuity design.

**Fig. 2:**
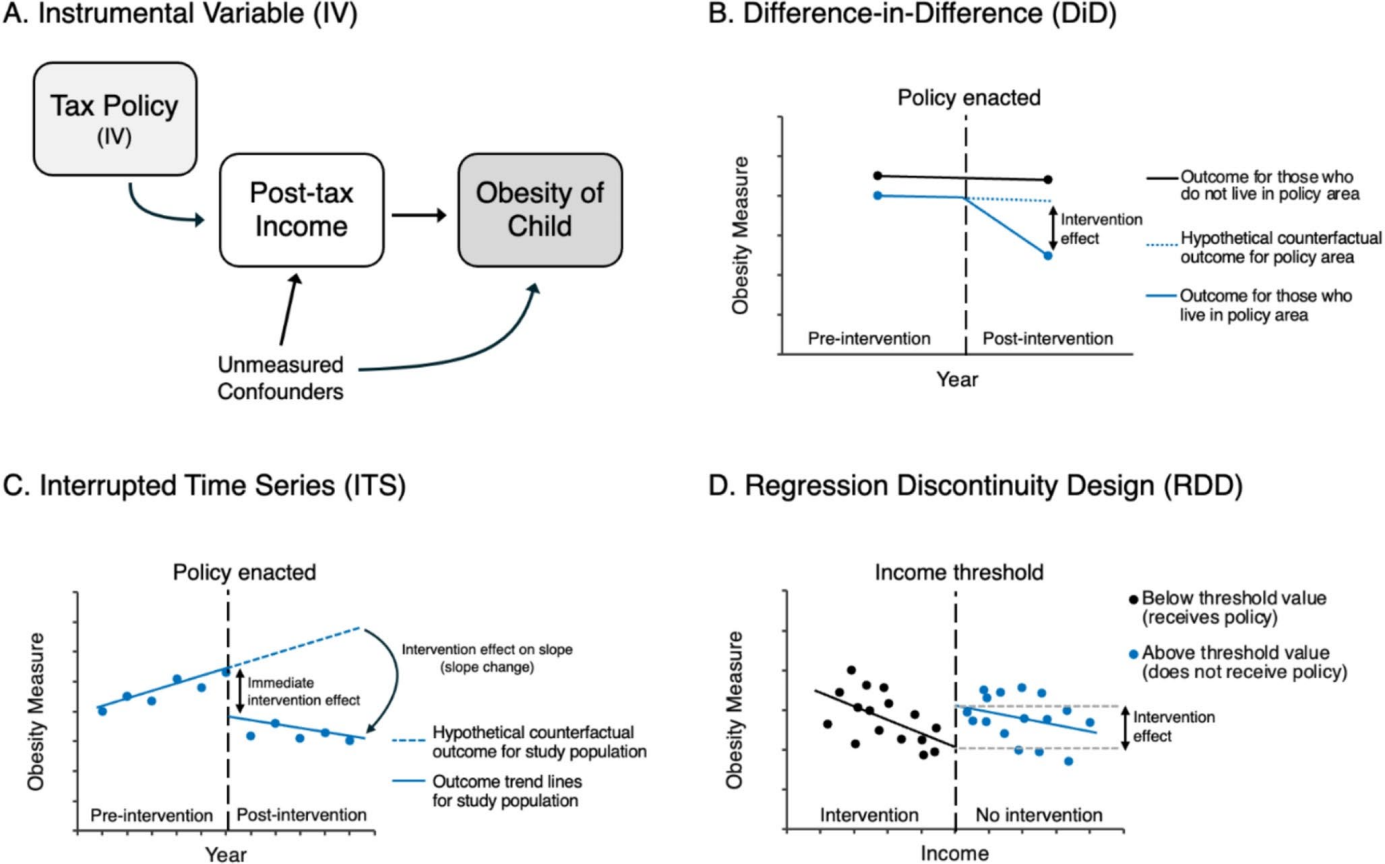
Quasi-experimental Study Designs to Investigate the Effect of Poverty Reduction on Childhood Obesity

Instrumental variable (IV) analysis is a causal inference method that originated in econometrics but has more recently been applied across disciplines including in epidemiology [80–82]. It utilizes a variable that is (i) associated with the treatment (relevance), (ii) affects the outcome only through the exposure (exclusion restriction), and (iii) does not share common causes with the outcome (exchangeability). For example, Jo leveraged the IV of a tax credit policy for low-income families to assess the association between after-tax income and the risk of childhood obesity [58]. The idea is that any increase in after-tax income would have come from the tax credit policy, and so any effects of increased after-tax income on childhood obesity outcomes should have flowed, causally, through the tax credit policy (Fig. 2a). The instrumental variable of tax credit policy has been used in other areas of health research as well [57].

Difference-in-differences (DiD) is another causal inference method that has been adopted into health research from the econometric literature [83] (Fig. 2b). A DiD analysis takes advantage of the time before and after a treatment (intervention or policy) is implemented to compare outcomes between the treated and untreated groups [84]. The key assumption that allows the difference between outcomes to be interpreted causally is that the difference between the treatment and control group would be constant over time in the absence of treatment, known as the parallel trend assumption. For example, Jones-Smith et al. used a DiD design to estimate the effects of opening or expanding a casino in American Indian tribal lands on childhood obesity outcomes [56]. The DiD design has been used in other studies of childhood obesity not limited to poverty as an exposure, such as supermarket access and universal school meal programs [85, 86]. In addition, staggered DiD methods have been increasingly developed in recent years that allow for consideration of multiple time periods and variations in the intervention timing [87].

Interrupted time series (ITS) analysis is another approach using multiple consecutive pre- and post-intervention observations (Fig. 2c). Instead of comparing the treatment and control groups, as in DiD, an interrupted time series analysis looks at the outcome trends in the time periods before and after the treatment in a single population [88, 89]. Under the assumption that the trend would remain constant in the absence of an intervention, the causal effect can be estimated by measuring the difference in trends, accounting for over-dispersion, autocorrelation, and time-varying confounders. For example, while the study by Chandran et al. did not examine poverty policy specifically, they used ITS and examined the effects of policy mandates to improve school lunch nutritional quality on decreases in children’s BMI [90].

One additional method that takes advantage of differences in receiving an intervention is regression discontinuity design **(**RDD) **(**Fig. 2d**)**. When a continuous variable is used to determine treatment assignment, RDD can be used to compare individuals (or other units of analysis) with values just above or below a specific cut-off threshold for treatment [91]. The idea is that those individuals just above or below the threshold value should have a similar distribution of background confounding factors, thus making them more comparable. The causal effect is estimated as the difference in outcome between those individuals above and below the treatment threshold. This approach is classified into sharp RDD (where the assignment variable deterministically dictates treatment) and fuzzy RDD (where the assignment variable influences the probability of receiving treatment). For instance, while Olsho et al. did not study childhood obesity as an outcome, they used RDD to investigate whether a program for low-income schools increased students’ intake of fresh fruits and vegetables [92]. The researchers examined dietary intakes among students who attended schools just above or below an income cut-off for their school to provide free fresh fruits and vegetables. While RDD is thought to have strong internal validity within the study sample, generalizability of results may be limited for individuals further from the threshold value [93].

### Questions of Causal Mechanisms

In addition to studying and answering questions of causality, many researchers and decision-makers may want to understand further the mechanisms between a given exposure and its outcome of interest. Mediation analysis can be used to parse out the direct and indirect effects of exposure on outcomes and can be applied to either interventional or observational data [94]. In the example of poverty and childhood obesity, a researcher may be interested in separating the direct effect of poverty on childhood obesity from the indirect effects mediated by factors such as lack of access to healthy food, exposure to fast food, and decreased physical activity environments. By distinguishing the indirect effects, one can quantify and assess the relative importance of a given mediating factor’s indirect effects on childhood obesity. While traditional mediation methods are based on adjusting for a mediating variable in a regression model to estimate a direct effect, they cannot account for nonlinear relationships and interactions between the exposure of interest and the mediator of interest [95]. In comparison, modern causal mediation analyses within the counterfactual framework are more flexible because they can account for such nonlinearity and exposure-mediator interactions under the required causal assumptions (e.g., conditional exchangeability, positivity, consistency, etc.) [94, 96].

**Fig. 3:**
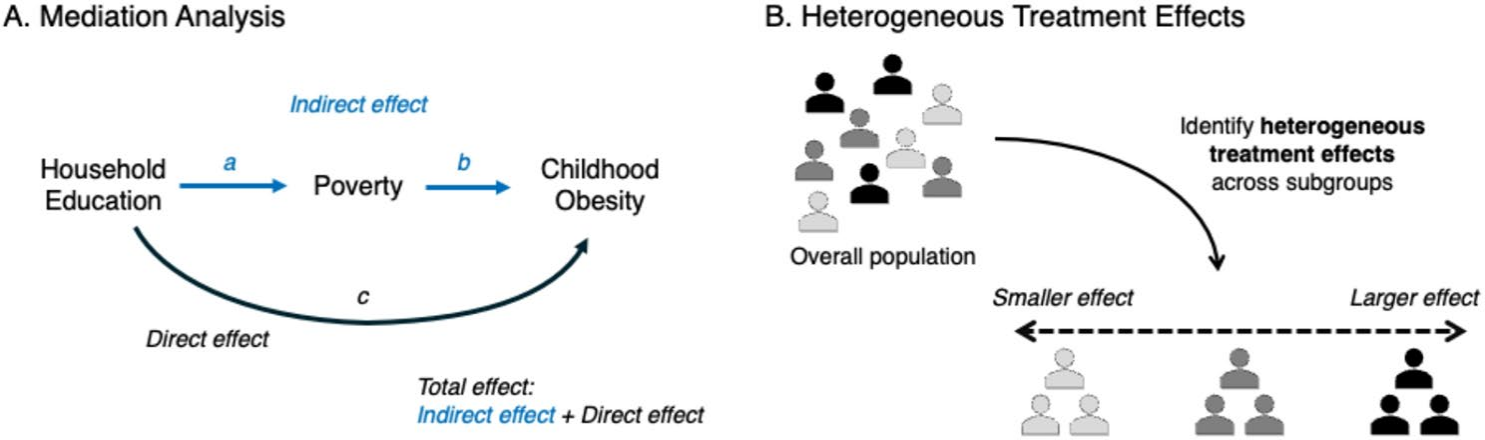
Methodologies to Study Mediating Pathways and Heterogeneity in Causal Effects

This approach of mediation analysis can also be applied to assess intergenerational disparities in childhood obesity (Fig. 3a). For example, using nationally representative data for US children and adolescents, Inoue et al. found that the current poverty status mediated around 20% of the association between low household education levels and childhood obesity [97]. Given that the past household education status is not modifiable, such quantification by mediation analysis provides practical insights into the effective strategies for improving situations caused by poverty, such as tax relief, cash transfers, and improving access to affordable healthcare, to reduce the intergenerational social disparity for childhood obesity.

### Heterogeneous Treatment Effects

Recent attention has been given to how interventions or exposures differentially affect health outcomes, known as heterogeneous treatment effects (HTE) (Fig. 3b). Although causal studies predominantly focus on estimating the average effect of treatments across populations, interventions such as social policies might impact the health outcomes of specific subpopulations differently [98]. Historically, subgroup analyses have been employed to investigate this heterogeneity [99]. With the rapid progression in computer science and the expansion of data, a variety of statistical and machine learning tools, including causal forest algorithms, have been designed to examine complex HTEs accounting for non-linear and high-order covariate interactions [100]. Applying these methods could enhance public health strategies by precisely targeting subpopulations that are most responsive to interventions [101] (e.g., cash transfers, medical interventions), or monitoring those most vulnerable to particular exposures [102] (e.g., poverty, natural disasters).

For example, previous studies using causal forest on data from the Oregon Health Insurance Experiment found the HTE in the improvements of blood pressure and mental health by health insurance coverage among low-income adults in the United States [103, 104]. These findings led to the identification of specific subgroups with large health benefits from health insurance coverage. While our literature search did not identify existing studies investigating HTE specific to childhood obesity, further work is essential for a deeper understanding of the heterogeneous links between poverty and childhood obesity and for designing policies that mitigate the health burden and social disparities of this preventable disease among children.

## Conclusion

Childhood obesity is a rising global health challenge, with major health and economic effects. Poverty reduction may reduce childhood obesity, though current evidence is mixed. The mixed results may be due to the multiple etiologies of childhood obesity, as well as the challenges of studying the effects of social policies. In addition to randomized controlled trials, the application of causal inference methods as outlined in this review may help provide new evidence on the extent to which poverty reduction decreases the risk of childhood obesity. Future studies and interventions should be clear as to what aspects of poverty they are targeting to better advocate and improve the health of children and their futures.

## Key References

Recently published papers of particular interest are highlighted as: • Of importance •• Of major importance.

••Goto R, Nianogo R, Okubo Y, Inoue K. Evaluation of Obesity Trends Among US Adolescents by Socioeconomic Status, 1999–2018. JAMA Pediatr. 2022;176:937.
  - Using the nationally representative data in the US, this study showed that socioeconomic disparities existed in obesity prevalence among US adolescents, and they have widened between 1999 and 2018. (Ref. 21)
•Dahabreh IJ, Bibbins-Domingo K. Causal Inference About the Effects of Interventions From Observational Studies in Medical Journals. JAMA. 2024;331:1845.
  - This special communication provides a practical framework for drawing causal inferences from observational data, crucial for studies on poverty and childhood obesity. This framework aims to enhance study quality and strengthen conclusions, thus providing more robust evidence on the link between poverty and childhood obesity. (Ref. 15)
••White PA, Awad YA, Gauvin L, Spencer NJ, McGrath JJ, Clifford SA, et al. Household income and maternal education in early childhood and risk of overweight and obesity in late childhood: Findings from seven birth cohort studies in six high-income countries. Int J Obes. 2022;46:1703–11.
  - This study found that low household income in early childhood is consistently linked to an increased risk of obesity later in childhood. The only exception was Sweden, where the effect size was smaller and the confidence interval crossed unity. Authors posit that this may be due to Sweden’s unique social and child health policies. (Ref. 24)
•Rummo P, Sze J, Elbel B. Association Between a Policy to Subsidize Supermarkets in Underserved Areas and Childhood Obesity Risk. JAMA Pediatr. 2022;176:646.
  - This paper demonstrates the use a quasi-experimental approach with observational data to investigate the effects of poverty-related policies on childhood obesity. The authors employ a difference-in-differences (DiD) analysis and creatively integrate both student-level and census tract–level data. (Ref. 81)
••Inoue K, Seeman TE, Nianogo R, Okubo Y. The effect of poverty on the relationship between household education levels and obesity in U.S. children and adolescents: an observational study. The Lancet Regional Health - Americas. 2023;25:100565.
  - Using causal mediation analysis, this cohort study quantified the extent to which poverty mediates the relationship between household education levels and childhood obesity. Such quantification help us to build effective strategies to mitigate the existing social disparity in obesity. (Ref. 93)

## Data Availability

No datasets were generated or analysed during the current study.
